# Ladies and Tobacco

**Published:** 1898-05-07

**Authors:** 


### The Hospital

A Journal of

The Medical Sciences and Hospital Administration.

Vol. XXIV.—No. 606.] Edited by Sir Henry Burdett, K.C.B., and Solomon C. Smith, M.D., M.R.C.P. [May 7, 1898.

### Ladies and Tobacco.

It is trite to observe that a large number of the most characteristic changes of the present rapidly culminating *fin de siècle* period have been in the direction of approximating the habits of the female to those of the male sex. Among the familiar illustrations of this fact, few are more remarkable than the increasing tendency of ladies towards the consumption of tobacco. In many foreign countries, of course, this is no novelty; and there are sunny lands in which the ashes of cigarettes form no unusual adornment of the keys of the pianoforte. In England, until a very recent period, the same thing could not be said; and the type of feminine smoker—or should it be smokeress?—was an old Irishwoman at a crossing, with a well-blackened clay pipe between her teeth. But time works its changes, and also reconciles us to them; with the consequence, among others, that London now contains clubs, some of which are open to lady members only, while others admit both ladies and gentlemen, and in both of them the question of the provision of a smoking-room for ladies has become what some of our contemporaries, without intending a joke, would call a "burning" one. Very much may be said upon either side of the question. Old-fashioned people are not unnaturally disposed to regard feminine smoking with a feeling which approaches horror, and to think that no woman can indulge in tobacco unless she be lost to all that makes her sex most charming, to delicacy, to refinement, to that power of self-denial for the sake of appearances that simulates, even if it does not constitute, much which passes muster for the highest order of virtue. The "new woman," on the other hand, does not see the object of denying herself any indulgence to which she has a fancy, and pitilessly scoffs at those who would restrain her. She regards them as prudes or as imbeciles. May there not be some middle aspect, and may there not be valid reasons why the use of tobacco is or is not desirable for either sex? Its influence, in all essentials, must be the same upon both, and the question whether it should be used must certainly be capable of elucidation, in the eyes of reasonable people, by an unprejudiced consideration of its effects.

We must begin by saying that we have not the smallest sympathy with that systematic consumption of tobacco which is only too widely spread among the younger males of the community, and which renders so many public places pestiferous. We think that the constant smoker among young men is very frequently one who is enabled by the perpetual influence of a narcotic to live in a fool's paradise, to neglect duties, to play with studies, to lose opportunities, and to whom it is quite certain that a bitter awakening will one day come. We are quite prepared to believe the correctness of the reports, which have come from certain American universities, of competitions between smokers and non-smokers, in which the former were beaten in everything—in games of skill, in trials of strength, and in position in the class-rooms. We are familiar, as every doctor must be, with the fact that many busy professional men, whose duties in life restrict their use of tobacco to certain periods of the day, and, therefore, to what is practically moderation, lose all the benefits which should naturally come from an autumnal holiday by smoking from morning to night during its continuance. But, when all this has been conceded, the fact remains that, to many persons, and according to the experience of the great majority of men folk, the moderate use of tobacco is a panacea against many of the smaller ills of life; and we think it cannot be denied that these smaller ills fall with far greater force, and with far more frequent recurrence, upon women than upon men. Why should women, if this be true, be deprived of the

> "Thought in the early morning, solace in time of woes,
> Peace in the hush of the twilight, balm ere the eyelids close."

We chance to know a London physician, who, whenever the soul of his wife has been troubled about any of the various household cares which beset the path of women, and from which it is so difficult for them to find escape, has gently urged upon her that she should seek the solace of tobacco, and so disarm her adversaries, who, for the most part, would be troublesome rather than really formidable. And this, we think, is advice which, in all probability, points to the true solution of the problem. Tobacco, like many other things, is "a good servant, but a bad master," and its excessive use, except in a few persons of exceptional temperament, tends to diminish the quantity, and to lower the quality of work. Its moderate use, on the other hand, again with the exception of a few persons to whom it is a poison, and with the still more important exception of the young, to whom it is probably always more or less prejudicial, tends to maintain the balance of the mind, to check despondency, to soothe irritation, and to bring about a greater fitness for the duties of daily existence. If this be true, it would surely be difficult to assign any valid reason why women should not smoke if they like to do so, and if they feel that they are helped by the process. Life is not so smooth that we should despise any contrivance for the diminution of its asperities or for the multiplication of its pleasures. Of all the follies into which the human race has fallen, surely the greatest and the worst has been asceticism; and it is only a survival of asceticism which could discover harmfulness in what to many is at once a benefit and a luxury.

